# Prunetin 4′-*O*-Phosphate, a Novel Compound, in RAW 264.7 Macrophages Exerts Anti-Inflammatory Activity via Suppression of MAP Kinases and the NFκB Pathway

**DOI:** 10.3390/molecules26226841

**Published:** 2021-11-12

**Authors:** Tae-Jin Park, Hyehyun Hong, Min-Seon Kim, Jin-Soo Park, Won-Jae Chi, Seung-Young Kim

**Affiliations:** 1 Department of Pharmaceutical Engineering & Biotechnology, Sunmoon University, Asan 31460, Korea; bark.taejin@gmail.com (T.-J.P.); kongjjo123@naver.com (H.H.); 2 Natural Product Informatics Research Center, Korea Institute of Science and Technology, Gangneung 25451, Korea; nari7040@gmail.com (M.-S.K.); jinsoopark@kist.re.kr (J.-S.P.); 3 Genetic Resources Assessment Division, National Institute of Biological Resources, Incheon 22689, Korea; wjchi76@korea.kr

**Keywords:** prunetin 4′-*O*-phosphate, prunetin, anti-inflammatory activities, biorenovation, phosphorylation, MAPK signaling

## Abstract

Biorenovation, a microbial enzyme-assisted degradation process of precursor compounds, is an effective approach to unraveling the potential bioactive properties of the derived compounds. In this study, we obtained a new compound, prunetin 4′-*O*-phosphate (P4P), through the biorenovation of prunetin (PRN), and investigated its anti-inflammatory effects in lipopolysaccharide (LPS)-treated RAW 264.7 macrophage cells. The anti-inflammatory effect of P4P was evaluated by measuring the production of prostaglandin-E_2_ (PGE_2_), nitric oxide (NO), which is an inflammation-inducing factor, and related cytokines such as tumor necrosis factor-α (TNFα), interleukin-1β (IL1β), and interleukin-6 (IL6). The findings demonstrated that P4P was non-toxic to cells, and its inhibition of the secretion of NO—as well as pro-inflammatory cytokines—was concentration-dependent. A simultaneous reduction in the protein expression level of pro-inflammatory proteins such as cyclooxygenase-2 (COX-2) and inducible nitric oxide synthase (iNOS) was observed. Moreover, the phosphorylation of mitogen-activated protein kinases (MAPKs) such as extracellular signal-regulated kinases (ERKs), c-Jun *N*-terminal kinase (JNK), p38 MAPK (p38), and nuclear factor kappa B (NFκB) was downregulated. To conclude, we report that biorenovation-based phosphorylation of PRN improved its anti-inflammatory activity. Cell-based in vitro assays further confirmed that P4P could be applied in the development of anti-inflammatory therapeutics.

## 1. Introduction

Under hazardous stimuli, for instance, infection and physical injury could trigger an inflammation response [1,2,3]. In particular, chronic inflammation could be harmful to normal tissues and cause many painful symptoms such as allergy, cardiovascular disease, diabetes, asthma, and cancer [4,5,6]. Therefore, inhibition of over-expressed inflammation is considered an effective strategy for treating inflammatory diseases [7,8].

Pathogenic bacteria-derived lipopolysaccharide (LPS) can be found on their extracellular surface, the presence of which activates macrophages and, in turn, induces the secretion of cytokines, such as tumor necrosis factor-α (TNFα), interleukin-1β (IL1β), and interleukin-6 (IL6) [9,10,11]. The increased secretion of cytokines promotes the gene and protein expression of inducible nitric oxide synthase (iNOS) and cyclooxygenase-2 (COX-2), as well as their respective end products nitric oxide (NO) and prostaglandin-E_2_ (PGE_2_) [12,13]. Reportedly, the iNOS-induced excessive expression of NO leads to cell death and disrupts tissue homeostasis [14,15].

Inhibitory κB (IκB) is normally bound to NFκB. During inflammation IκB kinase (IKK) phosphorylates IκB, and this allows NFκB to be released freely [16,17,18]. Simultaneously, the released NFκB is rapidly phosphorylated, which enables the translocation of NFκB to inside the nucleus through the nuclear membrane. DNA promoter-bound NFκB in turn facilitates the transcription and translation of inflammation-activating genes for COX-2, iNOS, IL6, IL1β, and TNFα [19,20]. LPS-induced mitogen-activated protein kinases (MAPKs) such as extracellular signal-regulated kinase (ERK), c-Jun *N*-terminal kinase (JNK), and p38 also play significant roles in NFκB activation [21,22,23,24]. Moreover, via these signaling pathways, transcription factors for the expression of inflammation proteins such as COX-2 and iNOS are activated. Accordingly, MAPKs and NFκB have been well studied over the development course of potential anti-inflammatory therapeutics. [25,26,27].

Flavonoids are secondary plant metabolites and are widely contained in fruits, vegetables, and certain drinks [28,29,30]. In addition, flavonoids are utilized in nutraceutical, pharmaceutical, medicinal, and cosmetic areas [31,32] and have a number of bio-activities, including anti-inflammatory, anti-lipogenic, and anti-cancer effects [33,34,35]. Flavonoids have a polyphenolic structure and consist of two main groups—2-phenylchromans and the 3-phenylchromans (isoflavones). Isoflavones include genistein, biochanin A, and formononetin, to name a few, and represent a portion of plant-derived secondary metabolites with estrogenic activity; therefore, they are known as phytoestrogens [36,37]. Of these, PRN (7-*O*-methylgenistein), an *O*-methylated isoflavone [38], has been shown to inhibit the gene expression and secretion of matrix metalloproteinase-3 (MMP)-3 in chondrocytes [39]. PRN has also been shown to suppress the transcription of obesity genes through a feedback mechanism [40].

Biorenovation facilitates the construction of a drug candidate library with biologically derivative natural compounds. It is a method of microbial enzyme-assisted biodegradation of natural compounds into novel bioactive candidates [41,42]. In our previous study, we showed its efficiency in improving the bioactivity of formononetin 7-*O*-phosphate derived from formononetin, and 4′-*O*-isopropyl genistein from genistein [43]. Given the multitude of bioactive properties of PRN, we hypothesized that biorenovation can improve the anti-inflammatory activities of PRN with no significant decrease in cell viability.

Therefore, in this study, we employed biorenovation to derivatize PRN and investigated the anti-inflammatory effects of the derived compound. Here, *Bacillus* sp. JD3-7 was used as a biocatalyst to derivatize PRN, and the biorenovation products were investigated for changes in their chemical structure and anti-inflammatory activities.

## 2. Results

### 2.1. Chemical Structures of the Biorenovation Products of PRN

**Figure 1:**
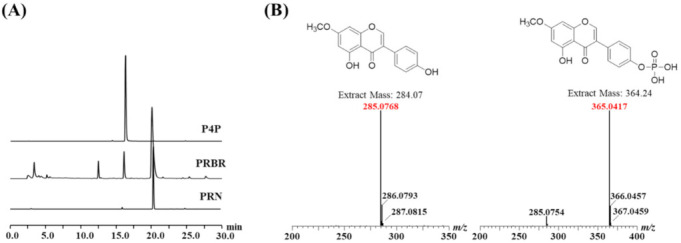
(**A**) HPLC analysis of prunetin (PRN) and the biorenovation products of prunetin (PRBR) and prunetin 4′-*O*-phosphate (P4P). (**B**) Mass analysis of PRN and P4P.

The biorenovation products of PRN (PRBR) using *Bacillus* sp. JD3-7 were detected using high-performance liquid chromatography (HPLC). Two different peaks, excluding PRN, were found in PRBR (Figure 1A).

The largest peak among the two, excluding the substrate, was purified using preparation HPLC. Electrospray ionization mass spectrometry (ESI/MS) was employed to ascertain whether the largest peak is a derivative of PRN. As shown in the mass spectrum in Figure 1B, a tallest peak at *m*/*z* 285 was identified as that of ionized PRN, and it showed that an [M + H^+^] peak at *m*/*z* 364 was that of a derivative of PRN. Based on previous studies [44], this result suggests that the hydrogen atom of PRN was phosphorylated by biorenovation.

### 2.2. NMR Results

The position of phosphorylation was confirmed by NMR experiments. The chemical shifts of H-3′ and H-5′ (δ_H_ 7.22 ppm) were downfield-shifted compared to that of prunetin. In addition, C-4′ was observed at 152.11 ppm in the ^13^C-NMR by shielding by phosphorylation. This result indicates phosphorylation at C-4′ of prunetin. C–P coupling with *J*_C-H_ 5.0 Hz caused a split in the carbon signals of C-3′ and C-5′ (^13^C-NMR spectrum). Through these results, the position of phosphorylation of P4P was elucidated.

(P4P, 1): ^1^H-NMR (DMSO-*d_6_*, 500 MHz): δ 8.40 (1H, s, C-2), 6.42 (1H, s, C-6), 6.68 (1H, s, C-8), 7.53 (2H, d, *J* = 3.5 Hz, C-2′, 3′), 7.22 (2H, d, *J* = 8.7 Hz, C-3′, 5′), 12.87 (1H, s, C5-OH), and 3.87 (1H, s, C7-OCH3) (Figure S1, Table S1).

^13^C-NMR (DMSO-*d_6_*, 125 MHz): δ 155.62 (C-2), 122.44 (C-3), 180.59 (C-4), 162.16 (C-5), 98.62 (C-6), 165.80 (C-7), 93.05 (C-8), 157.98 (C-9), 105.85 (C-10), 126.54 (C-1′), 130.58 (C-2′, 6′), 120.34 (d, *J* = 5.2 Hz, C-3′, 5′, 152.11 (C-4′), and 56.62 (C7-CH3) (Figures S2 and S3, Table S1).

### 2.3. Effects of P4P on the Viability of RAW 264.7 Cells and NO Production

**Figure 2:**
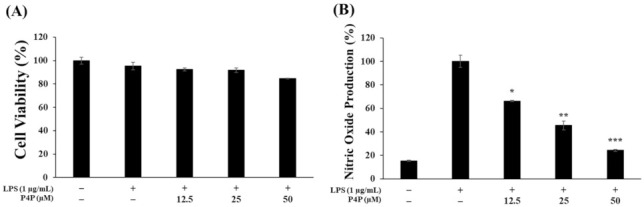
Activities of P4P on the toxicity and production of NO in RAW 264.7 cells induced by LPS. (**A**) Cell were treated with LPS (1 μg/mL) in the presence/absence of P4P (0.01% DMSO) for 24 h. (**B**) The secreted NO level among samples was compared. The values of triplicate experiments are represented as the mean and standard deviation (SD). * *p* < 0.05, ** *p* < 0.01, and *** *p* < 0.005 versus LPS alone.

The RAW 264.7 cells were treated with LPS only or with P4P of 12.5, 25, or 50 µM. As shown in Figure 2A, P4P did not significantly suppress the cells’ viability (Figure 2A). Furthermore, the inhibition of NO production was increased as with the increase in the P4P concentration (Figure 2B).

### 2.4. Suppression of LPS-Induced IL6, IL1β, and TNFα Production

**Figure 3:**
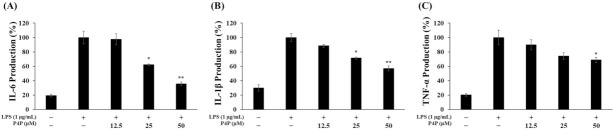
Effect of P4P on the (**A**) IL6, (**B**) IL1β, and (**C**) TNFα production in LPS-stimulated RAW 264.7 cells. Cell were treated with LPS (1 μg/mL) in the presence/absence of P4P (0.01% DMSO) for 24 h. Their production was determined by ELISA. The values of triplicate experiments are represented as the mean and standard deviation (SD). * *p* < 0.05 and ** *p* < 0.01 versus LPS alone.

The secreted cytokine levels were quantified with an ELISA assay, and their secretion levels among samples were compared. In the P4P-treated samples, IL6 production decreased by 3%, 38%, and 65% with the increase in P4P concentration of 12.5, 25, and 50 µM, respectively (Figure 3A). Similarly, P4P inhibited the expression of TNFα and IL1β concentration-dependently. Maximal inhibition on the production of IL1β and TNFα production was achieved at 50 μM of P4P by 43% and 31%, respectively (Figure 3B,C).

### 2.5. Suppression of LPS-Induced PGE_2_ Secretion and Protein Expression of iNOS and COX-2

**Figure 4:**
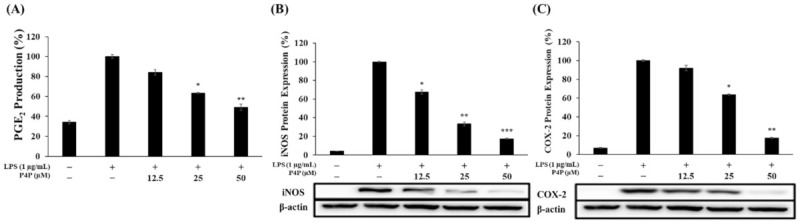
Inhibitory effects of P4P on LPS-induced PGE_2_ production and the protein levels of iNOS and COX-2 in RAW 264.7 cells. (**A**) Cell culture supernatant (100 μL) of cells stimulated with LPS (1 μg/mL) in the presence (12.5, 25, and 50 µM) or absence of P4P (0.01% DMSO) was used to determine the level of PGE_2_ production by ELISA. Absorbance was measured at 450 nm. (**B**,**C**) The protein levels of iNOS and COX-2 were determined by Western blot. * *p* < 0.05, ** *p* < 0.01, and *** *p* < 0.005 versus LPS alone.

LPS-induced PGE_2_ production was suppressed by 16%, 37%, and 51% with the increase in P4P concentration of 12.5, 25, and 50 µM, respectively (Figure 4A).

iNOS and COX-2 catalyze the production of NO and PGE_2_, respectively. Next, Western blotting was employed to examine the direct involvement of iNOS and COX-2 in their respective catalysis product of NO and PGE_2_. The two protein expression levels were significantly suppressed relative to LPS only, and P4P concomitantly decreased the protein levels of COX-2 and iNOS in a concentration-dependent manner. iNOS protein expression relative to LPS only was reduced by 67% and 83% with the increase in P4P concentration of 25 and 50 µM, respectively (Figure 4B). The reduction in COX-2 protein expression compared to LPS only was respectively decreased by 37% and 83% by P4P at 25 and 50 µM (Figure 4C). In addition, the iNOS and COX-2 proteins were downregulated to the levels of unstimulated RAW 264.7 cells. These results suggest the simultaneous downregulation of iNOS and COX-2 with their respective reduction in NO and PGE_2_ production.

### 2.6. Expression of Proteins of MAPK and NFκB Pathways

**Figure 5:**
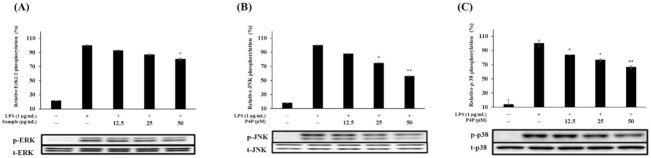

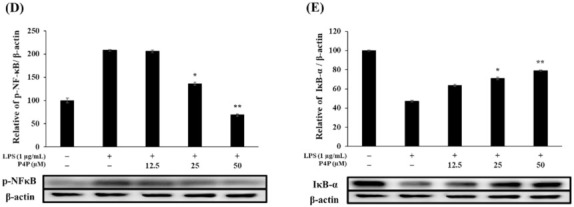
Effects of P4P on mitogen-activated proteins, including (**A**) ERK, (**B**) JNK, (**C**) p38, (**D**) NFκB, and (**E**) IκB-kinase activation. Cells were stimulated with LPS (1 µg/mL) in the presence (12.5, 25, or 50 µM) or absence of P4P (0.01% DMSO) for 40 min. the expression levels of MAPK, NFκB, IκB, and β-actin protein were measured. * *p* < 0.05, ** *p* < 0.01 versus LPS alone.

The presence of LPS in RAW 264.7 activates the MAPK (ERK, JNK, and p38) and NFκB signaling pathways to boost the production of inflammatory mediators and cytokines [45]. Western blot on the proteins of the MAPK and NFκB signaling proteins revealed that P4P suppressed the LPS-induced phosphorylation of ERK, JNK, and p38 and was downregulated with the increase in P4P concentration (Figure 5A–C).

Likewise, phosphorylation of p38 decreased with the increase in P4P concentration (Figure 5C). The results also indicate that P4P treatment decreased the level of NFκB phosphorylation (Figure 5D) while increasing the level of IκB-α expression (Figure 5E). Collectively, P4P exerted its anti-inflammatory properties by the downregulation of the MAPK and NFκB signaling proteins and hindering the secretion of related cytokines and mediators.

## 3. Discussion

Inflammation is an immune response in vivo that tries to defend the body and repair damaged areas when physical or chemical stimulations are applied to the body. During inflammation, macrophages produce inflammatory mediators (NO and PGE_2_) and related cytokines (IL6, IL1β, and TNFα). However, a persistent inflammatory response causes chronic inflammation, which, in turn, can damage body tissues or even lead to cancer [46]. In this respect, studies are being actively performed to find new substances that are active in inhibiting inflammation in order to prevent such chronic inflammation [47,48,49]. In addition, isoflavones such as genistein, daidzein, glycitein, formononetin, biochanin A, and prunetin are aglycone forms that lack glucose moieties. Recently, these low molecular weight isoflavones have been shown to exhibit diverse pharmacological properties such as anti-inflammatory, anti-oxidant, and anti-tumor effects [50,51,52,53]. Howerver, these isoflavones have setbacks such as poor hydrophilicity, which tends to prevent blood-borne drug delivery to inflamed sites and the solubilization of isoflavones in oral care product preparations. Using a high dose of isoflavones is also not recommended [54]. Thus, research to increase the water solubility of isoflavones is currently ongoing [55]. In a previous study, the 7-OH moiety of naringenin was replaced with a phosphate group using biotransformation technology, resulting in a 45-fold increase in the water solubility of naringein [56]. Accordingly, we employed biorenovation to improve the water solubility of isoflavones. In this study, we performed biorenovation using a known compound, PRN, and confirmed that the chemical structure converted to P4P, a novel compound, using MS and NMR. We then checked whether P4P has anti-inflammatory activity within the non-cytotoxic range, for which MTT and NO production tests were performed. In addition, the secretion levels of PGE_2_ and pro-inflammatory cytokines (IL6, IL1β, and TNFα), which are representative inflammation markers, were determined using an ELISA kit. Moreover, the protein levels of iNOS and COX-2, their respective catalyst for the production of NO and PGE_2_, were investigated through Western blot analysis. Our study confirmed that P4P exhibited its anti-inflammatory activities by the downregulation of inflammation signaling proteins and related cytokines.

The MTT test results confirmed that there was no cytotoxicity within the treated concentrations range (12.5, 25, and 50 μM), so subsequent experiments were carried out using these concentrations (Figure 2A). To determine whether P4P inhibits the production of inflammatory mediators at the following concentrations, RAW 264.7 macrophages were treated with LPS only or along with P4P sample treatments. As shown in Figure 2B and Figure 4A, both NO and PGE_2_ production were dramatically increased upon LPS treatment but were significantly decreased with an increasing concentration of P4P. In addition, we performed Western blot experiments to determine whether the reduction in these inflammatory mediators was regulated by the reduction in iNOS and COX-2 protein expression. As a result, the significantly attenuated expression of iNOS and COX-2 by P4P treatment was confirmed (Figure 4). Based on these results, it was confirmed that P4P treatment in LPS-stimulated macrophages downregulates both the protein expression of iNOS and COX-2 and their respective catalysis products NO and PGE_2_.

It has been reported that macrophages stimulated by LPS activate the initial inflammatory response by stimulating the production of pro-inflammatory cytokines [57]. Therefore, the effect of P4P on the expression of inflammation-related cytokines was evaluated with ELISA assays. As shown in Figure 3, it was confirmed that compared to LPS, only the level of cytokines (IL6, IL1β, and TNFα) was decreased with the increase in P4P concentrations.

The MAPK and NFkB signaling pathways are well-known in the regulation of the inflammatory response. Various harmful stimuli, such as LPS, ultraviolet rays, and chromosomal damaging substances, induce the activation of MAPK proteins (ERK, JNK, and p38) through their phosphorylation, which ultimately directly increases the production and expression of inflammation-related mediators and cytokines such as TNFα and IL1β. In addition, ERK is involved in signaling pathways that promote cell growth or differentiation [58,59,60]. To evaluate the inhibitory effect of P4P on the phosphorylation of the MAPK family, Western blot analysis was performed. The results showed that the significant downregulation of the LPS-induced phosphorylation of ERK, JNK, and p38 was observed with the increase in P4P concentration.

The NFκB signaling pathway is known to regulate the expression of iNOS and COX-2. The complex form of NFκB and IκB-α normally exists in the cytoplasm, but during an inflammatory response, NFκB phosphorylation and IκB-α degradation are promoted. In addition, phosphorylated NFκB translocates to inside nucleus, forming complexes with DNA promoters, increasing the transcription of inflammation-related genes such as for iNOS and COX-2, thereby promoting an inflammatory response [61,62]. Therefore, to confirm whether P4P affects phosphorylation in the NFκB signaling pathway, immunoblotting was conducted. The immunoblotting data show that P4P inhibited NFκB phosphorylation and IκB-α degradation in a concentration-dependent manner. Our mechanistic studies also suggest that P4P downregulated the expression of pro-inflammatory mediators and cytokines via the downregulation of the MAPK and NFκB signaling pathways.

In summary, this is the first time that biorenovation has been shown to modify the structure of PRN and to generate P4P with anti-inflammatory properties and increased aqueous solubility. The experimental results of LPS-treated RAW 264.7 macrophages show that P4P exerted the downregulation of inflammation in LPS-stimulated macrophages via the downregulation of the MAPK and NFκB signaling proteins. The water solubility of P4P is expected to increase, and our in vitro assays further confirmed that this could be utilized in the development anti-inflammatory therapeutics.

## 4. Materials and Methods

### 4.1. Chemical Preparation

Prunetin (PRN), trifluroacetic acid (TFA), acetonirile, 3-(4,5-dimethylthiazol-2-yl)-2,5-diphenyltetrazolium bromide (MTT), dimethyl sulfoxide (DMSO), Griess reagent (1% *w*/*v* sulfanilamide, 0.1% *w*/*v N*-(1-naphthyl)ethylene diamine hydrochloride, 2.5% *v*/*v* phosphoric acid), rabbit anti-mouse IgG antibody, and horseradish peroxidase (HRP) conjugate were purchased from Sigma-Aldrich (St. Louis, MO, USA). Nutrient broth containing peptones and beef extract was purchased from Oxoid (Waltham, MA, USA). The PG buffer (phosphate glycerin buffer) contained 2% *v*/*v* glycerin, 50 mM sodium phosphate, pH 7.2. Dulbecco’s modified eagle medium (DMEM), fetal bovine serum (FBS), a mixture of penicillin and streptomycin, and phosphate-buffered saline (PBS) were purchased from Gibco (Thermo Fisher Scientific, Inc., Waltham, MA, USA). FBS was inactivated at 56 °C for 30 min. DMEM was mixed with a heat-inactivated FBS (10% *v*/*v*) and antibiotics mixture (1% *v*/*v*). Skim milk was purchased from Becton (Dickinson & Co., Sparks, MD, USA).

### 4.2. Biorenovation of PRN

The strain used in the biorenovation was *Bacillus* sp. JD3-7 from the Korean Agricultural Culture Collection (designated number 92346P, KACC, Wanju, KOR). *B.* sp. JD3-7 was cultured in nutrient broth containing peptones and beef extract at 30 °C for 18 h. Afterward, the strains were pelleted by spin-down (4416 g, 15 min, 4 °C) to remove the supernatant, excluding pellets. The remaining cell culture media were completely washed out twice with PG buffer. Subsequently, PRN was cultured with PG buffer and *Bacillus* sp. JD3-7 microbes were added. The biorenovation was performed at 30 °C and 200 rpm for 72 h. Afterward, the biorenovation reaction was centrifuged at 4416× *g* for 10 min, and the debris-free liquid phase was freeze-dried.

### 4.3. HPLC Analysis and Purification of PRN Derivatives

High performance liquid chromatography (HPLC; Shimadzu, Shimadzu Scientific Instruments, Inc., Baltimore, DC, USA) was used to purify and analyze the PRN derivatives. HPLC consisting of a 3200 digital UV-VIS detector, controller, and Shim-Pack GIS C18, 5 µm, 250 × 4.6 mm I.D. (227-30106-08, Shimadzu Scientific Instruments, Inc., Baltimore, DC, USA). The column oven temperature was maintained at 40 °C. The binary solvent system consisted of (A) water with 0.1% *v*/*v* trifluoroacetic acid (TFA) and (B) acetonitrile (ACN), with a linear gradient of (B) 10%–100% ACN for 30 min. The PRN derivatives and PRN were injected at a 10 μL injection volume with a 1 mL/min flow rate.

### 4.4. LCMS and NMR Analysis

To decide the molecular weight of P4P and PRN, high-resolution quadrupole-time-of-flight electrospray ionization–mass spectrometry (HR-QTOF ESI-MS) consisting of UPLC coupled with a SYNAPT G2-Si column was achieved in positive ion mode. Using mass data analysis software, MassLynx ver. 4.1 (Waters Corporation, Milford, MA, USA), the masses for the PRN biorenovation products were extracted from the mass data and matched with the previously determined masses for the phosphorylated form of PRN. NMR spectra were obtained using an NMRS 500 NMR (Agilent Technology, Santa Clara, CA, USA) spectrometer with the residual solvent peaks (DMSO-*d6* = δH 2.50) of the deuterated NMR solvents as the reference peaks.

### 4.5. Cell Maintenance and Cytotoxicity Assays

RAW 264.7, mouse macrophage cell line (KCLB, Seoul, Korea), were cultured in DMEM (Gibco, Thermo Fisher Scientific, Inc., Waltham, MA, USA) supplemented with 10% *v*/*v* heat-inactivated fetal bovine serum (FBS; Gibco, Thermo Fisher Scientific, Inc., Waltham, MA, USA) and 1% *v*/*v* penicillin and streptomycin (Gibco, Thermo Fisher Scientific, Inc., Waltham, MA, USA). The cells were cultured in 18 cm^2^ culture plates (SPL Life Sciences Co., Ltd., Pocheon, Korea) at a density of 1 × 10^6^ cells/plate, maintained in a humidified environment at 37 °C and 5% CO_2_, and passaged every three days at approximately 70%–80% confluence to avoid overgrowth. Cells were seeded in 0.47 cm^2^ culture plates (Thermo Fisher Inc., Waltham, MA, USA) at a density of 8 × 10^4^ cells/well. LPS (1 µg/mL) alone and in combination with three different P4P concentrations of 12.5, 25, and 50 µM were added to each well at the same time and cultured for another 24 h. The level of cell detachment off the plate was quantified using MTT reagent [63]. The cells were incubated at a final concentration of 1 mg mL^−1^ of MTT in culture medium for 4 h in a CO_2_ incubator (Thermo Fisher Inc., Waltham, MA, USA) and then the spent culture medium was removed and washed twice in PBS. The insoluble form of formazan crystals was dissolved in DMSO, and its absorbance was determined on a microplate spectrophotometer (Multiskan GO, Thermo Fisher Inc., Waltham, MA, USA) at a 570 nm wavelength.

### 4.6. Quantification of NO and PGE_2_ Secretion

As described in Section 4.4., macrophages were stimulated with LPS (1 µg/mL) only or in combination with P4P (12.5, 25, or 50 µM) and further incubated another 24 h. The cultured cell supernatant (100 μL) was mixed with an equal volume of Griess reagent and incubated at room temperature for 10 min, and absorbance was measured at 540 nm to measure NO production [64,65]. PGE_2_ ELISA kits were purchased from R&D Systems (Minneapolis, MN, USA). In each case, sample measurements were performed in duplicate. The optical density was determined on a microplate spectrophotometer using a 450 nm wavelength.

### 4.7. Determination of IL6, IL1β, and TNFα Production

As described in Section 4.4., three concentrations of P4P (12.5, 25, and 50 µM) with or without LPS (1 µg/mL) were treated in macrophage cells. The inhibitory effect of P4P on the secretion of pro-inflammatory cytokines (IL6, IL1β, and TNFα) in the culture suspension was determined by ELISA kits, such as Mouse IL6 (Becton, Dickinson and Company, Sparks, MD, USA), Mouse IL1β/IL1F2 (R&D Systems, Minneapolis, MN, USA), and Mouse TNF alpha (Invitrogen, Carlsbad, CA, USA).

### 4.8. Immunoblotting

Immunoblotting was carried out as per the method of Kocic et al. [66]. RAW 264.7 cells were lysed in RIPA buffer (Bio-Rad, Hercules, CA, USA) containing protease inhibitor. A Bradford assay kit (Pierce BCA Protein Assay Kit, Thermo Scientific, Waltham, MA, USA) was used to quantify the extracted proteins. After protein molecular weight (20 µg)-based separation by 10% SDS-PAGE, the resultant gel was blotted to 0.45 µm polyvinylidene difluoride (PVDF) membranes (Bio-Rad, Hercules, CA, USA) using the Trans-Blot Turbo system (Bio-Rad, Hercules, CA, USA). The blotted membranes were then blocked with 10% *w*/*v* skim milk for 2 h and washed four times with TBST (Tween 20-added TBS at 0.05% *v*/*v*). Afterward, the membranes were incubated overnight at 4 °C with primary antibodies such as COX-2, iNOS, Phospho-Erk1/2 (Thr202/Tyr204), Phospho-p38 (Thr180/Tyr182), Phospho-SAPK/JNK (Thr183/Tyr185), p44/42 MAPK, p38, SAPK/JNK, Phospho-NFkB p65 (Ser536) (93H1), and IκB-α (L35A5). Then, the membranes were conjugated with secondary antibody (rabbit anti-mouse IgG antibody) at room temperature for 2 h, and the antibody-bound proteins on the membranes were visualized using enhanced chemiluminescence reagent (ECL) (Bio-Rad, Hercules, CA, USA). Additionally, the presence and intensity of the target protein on a membrane were measured using ImageQuant 1.3 (LAS4000 mini, GE Healthcare Japan Corp., Tokyo, Japan).

### 4.9. Statistical Analyses

The measured values are represented as the mean ± standard deviation. Statistically significant differences between means were uncovered using unpaired Student’s *t*-tests (95% confidence).

## Data Availability

Not applicable.

## Supplementary Materials

The following are available online, Figure S1: ^1^H-NMR spectra of prunetin 4′-*O*-phosphate (P4P, 1); Figure S2: ^13^C-NMR spectra of prunetin 4′-*O*-phosphate (P4P, 1); Figure S3: HMBC correlation of prunetin 4′-*O*-phosphate (P4P, 1); Table S1: ^1^H and ^13^C-NMR chemical shifts of prunetin 4′-*O*-phosphate (P4P, 1).

## Sample Availability

Samples of the compounds aren’t available from the authors.
