# Supplementary material for: Prunetin 4′-O-Phosphate, a Novel Compound, in RAW 264.7 Macrophages Exerts Anti-Inflammatory Activity via Suppression of MAP Kinases and the NFκB Pathway
Source: Molecules. 2021 Nov 12;26(22):6841. doi: 10.3390/molecules26226841 (PMC8622051; doi:10.3390/molecules26226841)
Supplement: Supplementary file 1 [file molecules-26-06841-s001.zip › molecules-1417692-supplementary.pdf]

molecules
MDPI

Article

Prunetin 4′-*O*-Phosphate, a Novel Compound, in RAW 264.7 Macrophages Exerts Anti-Inflammatory Activity via Suppression of MAP Kinases and the NFκB Pathway

Taejin Park [1], Hyehyun Hong [1], Min-Seon Kim [2], Jin-Soo Park [2], Won-Jae Chi [3] and Seung-Young Kim [1,*]

[1] Department of Pharmaceutical Engineering & Biotechnology, Sunmoon University, Asan 31460, Korea; bark.taejin@gmail.com (T.P.); kongjjo123@naver.com (H.H.)
[2] Natural Product Informatics Research Center, Korea Institute of Science and Technology, Gangneung 25451, Korea; nari7040@gmail.com (M.-S.K.); jinsoopark@kist.re.kr (J.-S.P.)
[3] Genetic Resources Assessment Division, National Institute of Biological Resources, Incheon 22689, Korea; wjchi76@korea.kr
* Correspondence: sykim01@sunmoon.ac.kr; Tel.: +82-41-530-2390

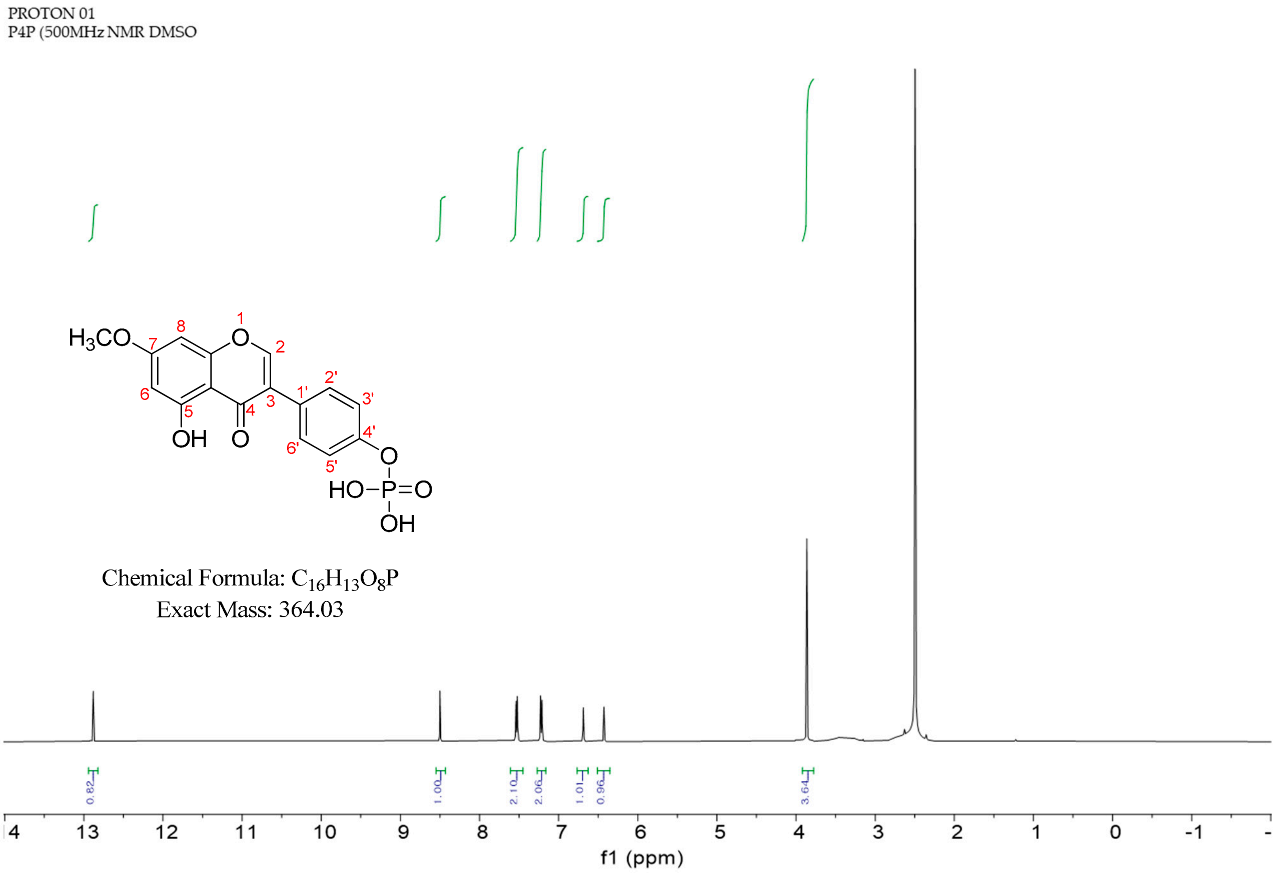

Figure S1. ^{1}H-NMR spectrum of prunetin 4′-*O*-phosphate (P4P, 1).

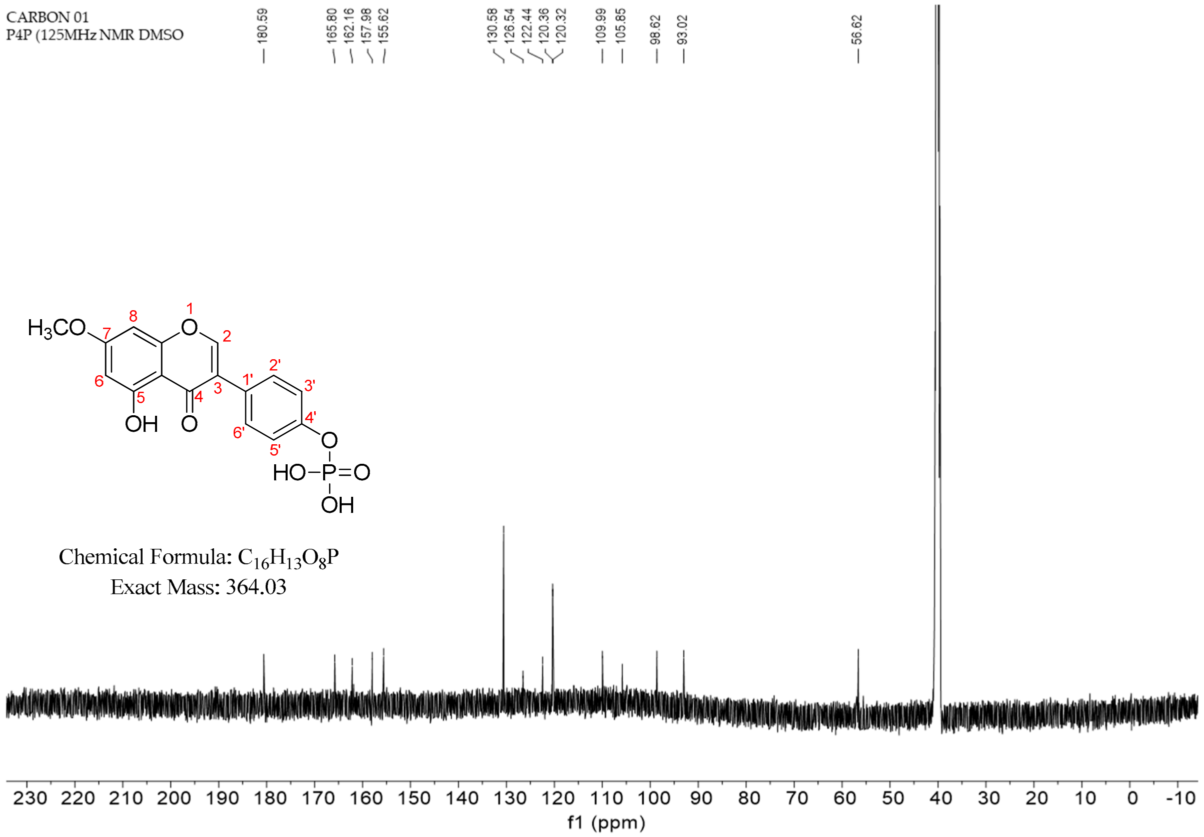

Figure S2. ^{13}C-NMR spectrum of prunetin 4′-*O*-phosphate (P4P, 1).

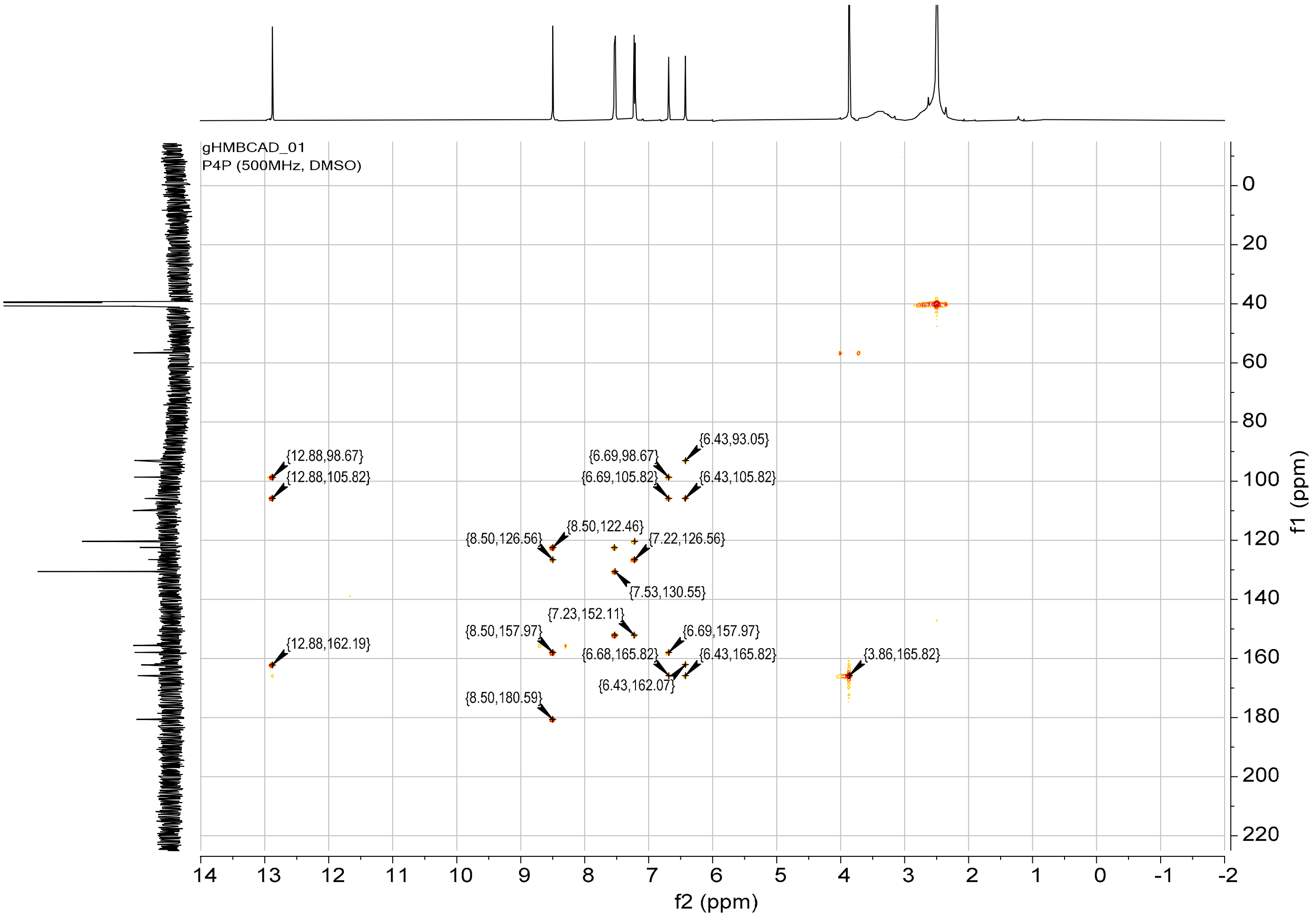

Figure S3. HMBC correlation of prunetin 4′-*O*-phosphate (P4P, 1).

Table S1. ^{1}H (DMSO-*d*6, 500 MHz NMR) and ^{13}C (DMSO-*d*6, 125 MHz NMR) NMR chemical shifts of prunetin 4′-*O*-phosphate (P4P, 1).

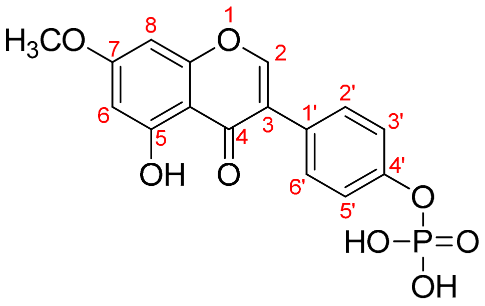

Compound	No.	[1]H	[13]C
Chemical Formula: $C_{16}H_{13}O_8P$<br>Exact Mass: 364.03	2	8.40 s	155.62
	3		122.44
	4		180.59
	5		162.16
	6	6.42 d	98.62
	7		165.8
	8	6.68 d	93.05
	9		157.98
	10		105.85
	1′		126.54
	2′ and 6′	7.53 d	130.58
	3′ and 5′	7.22 d	120.34 d
	4′		152.11
	5-OH	12.87 s	
	7-OCH_3	3.87 s	56.62
